# Is there a role of statins in the prevention of aortic biological prostheses degeneration

**DOI:** 10.1186/1476-7120-4-26

**Published:** 2006-06-29

**Authors:** Francesco Antonini-Canterin, Alfredo Zuppiroli, Ferdinando Baldessin, Bogdan A Popescu, Gian Luigi Nicolosi

**Affiliations:** 1 Unità Operativa di Cardiologia, A.R.C. Azienda Ospedaliera S. Maria degli Angeli, Pordenone, Italy; 2 U.O. Cardiologia, Ospedale S. Maria Annunziata, Azienda Sanitaria 10, Firenze, Italy; 3 Institute of Cardiovascular Diseases, Bucharest, Romania

## Abstract

It has been recently observed that statins might slow the progression of aortic stenosis or sclerosis. Preliminary reports suggested a similar positive effect in reducing the degeneration of aortic valve bioprostheses even though this hypothesis should be further proven and supported by new data. In this review the present evidences of the possible effects of statins in this field are discussed.

## Review

Nowadays many aortic valve prostheses are available in clinical practice: mechanical, biological and human tissue prostheses. None of the currently available prosthetic valves, however, approach the human valve in terms of hemodynamic function and/or freedom from complications. Mechanical prostheses have a longer durability and provide a generally good hemodynamic function, but are thrombogenic, requiring permanent anticoagulation with related risks in terms of morbidity and mortality [1,2]. On the contrary, bioprostheses have a low thrombogenicity and do not require anticoagulation. On the other hand, bioprosthetic valves have the propensity to undergo structural degeneration, limiting their durability and often necessitating reoperation. For these reasons bioprosthetic valves are usually used in old patients, while mechanical ones are preferred in young people. In any case, it should be recognized that the choice in the individual patient is not simple.

Aortic valve replacement is one of the most frequently performed cardiac surgery interventions in western countries and the proportion of biological valves used in the aortic position can be as high as 70% in patients over 70 years [3,4]. Moreover, given the increased age at implantation of the commonly referred patients, the proportion of biological prostheses is increasing. Thus, a medical therapy able to reduce bioprosthetic valve structural degeneration would have an important clinical and socio-economic impact.

It has been recently observed that hydroxymethylglutaryl coenzyme A reductase inhibitors might slow the progression of mild and moderate stenosis or sclerosis in native aortic valves [5-7]. Several studies suggested that atherosclerosis and aortic valve stenosis could be considered simply different manifestations of the same disease [8-11]. In a cholesterol-fed rabbit model, hypercholesterolemia induced atherosclerotic-like lesions in the aortic valve tissue, and atorvastatin reduced this phenomenon [12]. Furthermore, a number of retrospective, non-randomized studies reported a possible effect of statins in slowing progression of mild or moderate aortic stenosis in human native valves [5-7]. Other studies [13,14] using electron-beam computed tomography demonstrated a decreased rate of aortic valve calcium accumulation in statin-treated patients. However, the real role of hydroxymethylglutaryl coenzyme A reductase inhibitors is not yet clarified. The correlation between cholesterol levels and aortic stenosis progression is still controversial. Some studies [5,13] found a significant correlation, while other ones [6,7] showed lack of correlation with aortic stenosis progression. Also the hypothesis that statin benefits could be due to their pleiothropic and anti-inflammatory properties has yet to be demonstrated.

In addition, the last two studies on this topic, published this year, did not confirm the positive effect of hydroxymethylglutaryl coenzyme A reductase inhibitors on aortic stenosis progression (Table 1) [15,16]. Cowell et al. [15] assessed aortic valve stenosis and calcification progression with Doppler echocardiography and computed tomography, in a prospective double-blind-controlled trial; patients were randomly assigned to receive either atorvastatin or placebo. Their conclusion was that intensive lipid-lowering therapy does not halt the progression of calcific aortic stenosis or induce its regression. Our group [16] demonstrated a positive effect of statins only in the subgroup of patients with aortic valve sclerosis, suggesting that these drugs could be effective only in the early phase of disease.

**Table 1:** Aortic valve stenosis progression.

**Author (year of publication) [reference]**	**N. patient (receiving statins)**	**Follow-up (months)**	**Mean age (years)**	**Prospective/retrospective**	**Randomized/non randomized**	**CAD (%)**	**A. Vel. max (m/sec)**	**Positive effect of statin**
**Aronow et al. (2001) [5]**	180 (62)	33	82 ± 5	retrospective	non-randomized	NA	NA	Yes
**Novaro et al. (2001) [6]**	174 (57)	21	68 ± 12	retrospective	non-randomized	59	2,65	Yes
**Bellamy et al. (2002) [7]**	156 (38)	44	77 ± 12	retrospective	non-randomized	35	2,95	Yes
**Rosenhek et al. (2004) [26]**	211 (50)	24	70 ± 10	retrospective	non-randomized	27	3,96	Yes
**Antonini-Canterin et al. (2005) [25]**	242 (121)	48	67 ± 9	retrospective	non-randomized	46	2,45	No
**Cowell et al. (2005) [15]**	134 (65)	25	68 ± 11	prospective	randomized	20	3,42	No

Authors of the studies with years follow-up, mean age, cardiovascular risk factors, peak aortic velocity and the presence of a positive effect of statins on aortic valve stenosis progression.

The problem of early calcification of bioprosthetic valves is well known. Indeed, at the time of commercial manufacturing [17], a treatment with the T6 (a detergent sodium dodecyl sulphate to retard calcification [18]) is usually performed to remove the lipids from the porcine valve. Nevertheless, it cannot prevent subsequent lipid insudation that may favor calcification. Anyway, with T6 mitigation, dystrophic calcification may be delayed until other factors come into play. Lipid insudation and monocyte infiltrates occur in the cuspidal tissue of porcine bioprostheses as seen in early atherosclerosis and can precipitate structural valve deterioration in the long-term, even in the absence of mineralization [19]. Bottio et al. hypothezised that lipids could play a role in the structural valve deterioration of bioprostheses. In their study they observed, by electron microscopy, lipid insudations in almost all the explanted biological valves [20].

Farivar and Cohn [21] have recently suggested that hypercholesterolemia could be considered a risk factor for bioprosthetic valve calcification and explantation. They performed a retrospective cohort study on 144 patients who had bioprosthetic aortic or mitral valves removed. In a subgroup of 66 patients they performed a case-control analysis of the tissue valves explanted and compared them with an age- and position-matched group of 66 patients with similar duration of implantation. The mean serum cholesterol level in the explanted valve group was significantly higher (189 vs 163 mg/dL, p < .0001) than that of the group whose valves did not require explantation. This supports the potential role of hypercholesterolemia as a risk factor for bioprosthetic valve calcification requiring explantation. Similar results were found by Nollert et al. [22]. In these two studies both aortic and mitral bioprostheses were evaluated, but the role of statin treatment was not addressed to.

David and Ivanov [23], analyzing two large databases from Stanford University and Toronto General Hospital, did not confirm a role of hyperlipidemia in predicting freedom from reoperation after aortic valve replacement with bioprosthetic valves. However, the authors could not exclude the hypothesis that the probability of valve failure in patients with risk factors for atherosclerosis was reduced because most of them were actually taking statins.

**Figure 1:**
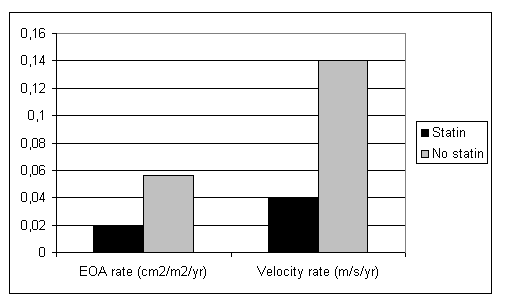
Peak Prosthetic Velocity and Prosthetic Indexed Effective Orifice Area. Comparison of Annual Rate of increase in Peak Prosthetic Velocity (m/s/yr) and annual rate of change in Prosthetic Indexed Effective Orifice Area (EOA) (cm2/m2/yr) in patients with statins therapy and those without.

Our group was the first one to observe a positive effect of statins on reducing the progression of bioprosthetic aortic valve degeneration [24,25]. In order to assess whether statins play a role in slowing degeneration of bioprosthetic aortic valves we have retrospectively selected from our 15-years database (1988–2002) all the patients with bioprosthetic aortic valves having at least 2 echocardiographic examinations at least 6 months apart. There were 167 patients (97 men, mean age 71 ± 9 years at the first examination), followed for 46 ± 38 months. During follow-up, 22 patients (13%) were treated with statins, while 145 (87%) were not. There were no differences between the two groups regarding age, gender, follow-up duration, baseline peak aortic velocity, mean gradient, effective orifice area, degree of aortic regurgitation, and left ventricular ejection fraction. As expected, statin-treated patients had a significantly higher prevalence of documented hypercholesterolemia, proven coronary artery disease, and associated coronary artery bypass surgery (p < 0.001 for each). There were no significant differences between the two groups in prosthetic size, or prosthetic type (stented vs stentless, or porcine vs pericardial valves). The annual rate of increase in peak prosthetic velocity was lower in statin-treated patients (0.038 ± 0.074 vs. 0.140 ± 0.228 m/sec/year, p < 0.001). The annual rates of decrease in prosthetic effective orifice area (0.031 ± 0.052 vs 0.100 ± 0.150 cm^2^/year) and indexed effective orifice area (0.019 ± 0.031 vs 0.056 ± 0.086 cm^2^/m^2^/year) were also lower in statin-treated patients (p < 0.001 for both) (Figure 1). Worsening of aortic regurgitation was found in 2/22 (9.1%) in the statin group and in 48/145 (33.1%) of controls (p = 0.022). The existence of either a rate of increase in peak velocity ≥ 0.3 m/s/year or worsening of aortic regurgitation ≥ 1/3 degree was found in 2/22 (9.1%) of statin-treated and in 63/145 (43.4%) of non-treated patients (p = 0.002) (Odds ratio with statin treatment: 0.13; 95% CI, 0.03–0.58). The overall annual rate of progression in peak prosthetic velocity was similar between porcine and pericardial valves and between stented and stentless valves. The only factor associated with a lower progression of bioprosthetic aortic valve failure was statin treatment. During follow-up, there was no difference in major clinical event occurrence between the two groups. No significant adverse effects of statin treatment were recorded during follow-up. Our study was the first one to provide evidence that statin treatment is associated with significantly less bioprosthetic aortic valve failure, opening a new field for clinical research. It should be recognized, however, that there are several limitations: given the small number of patients with different types of bioprostheses on statins, a meaningful subgroup analysis of the differences in outcome between different types of biological prostheses was not possible. Because of the retrospective nature of the study and because of the inclusion period, complete information regarding lipid profile was not available. Therefore, we could not test for a relation between changes in lipid profile and bioprosthetic aortic valve degeneration, and so the mechanism of statin treatment benefit in this setting remains speculative.

Rosenhek et al. [26], in a preliminary report, did not confirm the positive effect of hydroxymethylglutaryl coenzyme A reductase inhibitors on aortic valve bioprostheses. A possible explanation for a lack of effect of statin treatment in this study is the rather short follow-up (28 ± 17 months), as it would be sound to expect a benefit of statin treatment in this setting after a longer period. Another explanation could be the use of only peak transprosthetic velocity and lack of inclusion of aortic regurgitation as a measure of hemodynamic deterioration of bioprosthetic aortic valves in their study. In two studies, Skowasch et al. have recently supported the hypothesis of the inflammatory process affecting aortic valve bioprostheses and the pleiotropic effects of statins in these patients. In one study [27], they analyzed the endstage degenerative aortic valve tissue from native valve of 57 consecutive patients, tissue from aortic porcine degenerated bioprostheses of 24 patients and tissue from 5 non-stenosed native control valves; the serum C-reactive protein levels were also measured preoperatively. They found C-reactive protein more frequently in bioprostheses than in native valves. Also the serum C-reactive protein levels increased in patients with aortic valve bioprostheses showing a significant correlation with the valvular inflammatory process. Valvular C-reactive protein expression and serum C-reactive protein levels were found to be lower in the group of patients with a statin treatment than in the group without it suggesting possible pleiotropic and/or anti-inflammatory properties of these molecules. In another study [28], the same authors analyzed aortic valves from patients with non-rheumatic aortic valve stenosis and with degenerative aortic valve bioprostheses searching for the presence of endothelial progenitor cells and leukocyte subtype-specific markers. These cells were detected in a large series of degenerative aortic valves, more frequently in bioprostheses than in native cusps. These findings suggest not only a unifying pathogenic mechanism that underlies both types of valvular degeneration, but also an even more important role of primarily extravalvular cells in the case of prosthesis degeneration. In this work on high-grade aortic stenosis, a significant relationship between endothelial progenitor cell markers and statins, aspirin or ACE-inhibitors was not found.

Aortic valve prostheses most often behave hemodynamically like a mildly stenotic native valve and the pattern of flow through the valve is similar. This mechanistic similarity of the hydrodynamic patterns could be another explanation for a similar benefit of statins treatment in both native and bioprosthetic aortic valves. On the other hand, statins exhibit pleiotropic effects over and above lipid lowering, including anti-inflammatory effects [29]. They retard extra-osseous calcifications, as for coronary vessels [30], and decrease native aortic valve calcium accumulation [13,14]. Of note, doses used in statin-treated patients in our study were relatively low compared to currently used dosages. Because of the submaximal doses the actual effect of statins might have been underestimated. It is also noteworthy that statin-treated patients had a reduced progression of bioprosthetic degeneration despite a higher number of risk factors.

Wu et al. [31] recently evaluated the effect of statins on aortic valve myofibroblasts and osteoblast calcification in vitro. Interestingly, statins inhibited calcification in aortic valve myoblasts but paradoxically they stimulated bone cell calcification in valve osteoblasts. At implantation, bioprosthetic aortic valves represent "de novo" structures, not yet affected by degenerative calcific process. Theoretically, statins could be more effective in this subgroup of patients with bioprotheses as compared to the general aortic native valve population, where the degenerative process has already started and osteoblasts are likely to be activated by statins.

Martinez-Gonzalez et al. [32] showed that human and porcine smooth muscle cells share similar proliferation dependence on the mevalonate pathway, inhibited by statins treatment. They concluded that the porcine model closely resembles the human model and it may be suitable for testing new treatment strategies in vivo. More recently, the same authors showed a positive effect of statin treatment on vessel wall expression of a protein involved in atherosclerotic lesion progression in a hypercholesterolemic porcine model [33]. This could explain in part a similar positive effect of statins in human and porcine valves.

In conclusion, treatment with statins could be associated with significantly less degeneration of bioprosthetic aortic valves, but a definitive proof is still awaited. The data from Cowell's study support the hypothesis that intensive lipid-lowering therapy doesn't slow the progression of the aortic stenosis of the native valve; but the same author reinforce the need for a long-term and large-scale trial because these were the limits of his investigations. The ASSIST study (Asymptomatic aortic Sclerosis/Stenosis: Influence of STatins), an ongoing study of the "Società Italiana di Ecografia Cardiovascolare", is aiming to create a large, prospective, observational investigation, involving many Echocardiograpic laboratories and thousands of patients, in order to give more definite answers from the real clinical world to this unsolved question. A substudy of the ASSIST study on aortic biological prostheses is also planned, in order to prospectively assess, in a large number of patients, the potential role of this pharmacologic treatment in reducing the rate of prostheses degeneration.

## Conclusion

The potential role of statin treatment in reducing the rate of aortic biological prostheses degeneration has not been clarified yet. Nowadays studies are discordant but a large, prospective, observational investigation is lacking. On going studies could answer some of the unsolved questions.

## Abbreviations

None

## Competing interests

The author(s) declare that they have no competing interests.
